# Development and Characterization of a Highly Sensitive NanoLuciferase-Based Immunoprecipitation System for the Detection of Anti-Influenza Virus HA Antibodies

**DOI:** 10.1128/mSphere.01342-20

**Published:** 2021-05-12

**Authors:** Tomoko Honda, Sumiko Gomi, Daisuke Yamane, Fumihiko Yasui, Takuya Yamamoto, Tsubasa Munakata, Yasushi Itoh, Kazumasa Ogasawara, Takahiro Sanada, Kenzaburo Yamaji, Yasuhiro Yasutomi, Kyoko Tsukiyama-Kohara, Michinori Kohara

**Affiliations:** a Department of Microbiology and Cell Biology, Tokyo Metropolitan Institute of Medical Science, Tokyo, Japan; b Laboratory of Immunosenescence, National Institutes of Biomedical Innovation, Health and Nutrition, Osaka, Japan; c Division of Pathogenesis and Disease Regulation, Department of Pathology, Shiga University of Medical Science, Setatsukinowa, Otsu, Shiga, Japan; d Tsukuba Primate Research Center, National Institutes of Biomedical Innovation, Health and Nutrition, Ibaraki, Japan; e Transboundary Animal Diseases Centre, Joint Faculty of Veterinary Medicine, Kagoshima University, Kagoshima, Kagoshima, Japan; University of Maryland School of Medicine

**Keywords:** NanoLuciferase (NLuc), broad dynamic range, cynomolgus macaque, influenza virus hemagglutinin (HA) protein, luciferase immunoprecipitation system (LIPS), mouse, tree shrew, trimeric complex

## Abstract

Antibody detection is crucial for monitoring host immune responses to specific pathogen antigens (Ags) and evaluating vaccine efficacies. The luciferase immunoprecipitation system (LIPS) was developed for sensitive detection of Ag-specific antibodies in sera from various species. In this study, we describe NanoLIPS, an improved LIPS assay based on NanoLuciferase (NLuc), and employ the assay for monitoring antibody responses following influenza virus infection or vaccination. We generated recombinant influenza virus hemagglutinin (HA) proteins tagged with N-terminal (N-NLuc-HA) or C-terminal (C-NLuc-HA) NLuc reporters. NLuc-HA yielded an at least 20-fold higher signal-to-noise ratio than did a LIPS assay employing a recombinant HA-Gaussia princeps luciferase (GLuc) fusion protein. NanoLIPS-based detection of anti-HA antibodies yielded highly reproducible results with a broad dynamic range. The levels of antibodies against C-NLuc-HA generated by mice vaccinated with recombinant vaccinia virus DIs strain expressing an influenza virus HA protein (rDIs-HA) was significantly correlated with the protective effect elicited by the rDIs-HA vaccine. C-NLuc-HA underwent glycosylation with native conformations and assembly to form a trimeric structure and was detected by monoclonal antibodies that detect conformational epitopes present on the globular head or stalk domain of HA. Therefore, NanoLIPS is applicable for evaluating vaccine efficacy. We also showed that C-NLuc-HA is applicable for detection of HA-specific antibodies in sera from various experimental species, including mouse, cynomolgus macaque, and tree shrew. Thus, NanoLIPS-based detection of HA offers a simple and high-sensitivity method that detects native conformational epitopes and can be used in various experimental animal models.

**IMPORTANCE** Influenza virus HA-specific antibodies can be detected via the hemagglutination inhibition (HI) assay, the neutralization (NT) assay, and the enzyme-linked immunosorbent assay (ELISA). However, these assays have some drawbacks, including narrow dynamic range and the requirement for large amounts of sera. As an alternative to an ELISA-based method, luciferase immunoprecipitation system (LIPS) was developed. We focused on NanoLuciferase (NLuc), which has a small size, higher intensity, and longer stability. In this study, we developed a technically feasible and highly sensitive method for detecting influenza virus-specific antibodies using a NLuc-tagged recombinant HA protein produced in mammalian cells. HA with a C-terminal NLuc extension (C-NLuc-HA) was glycosylated and formed trimeric complexes when expressed in mammalian cells. Furthermore, C-NLuc-HA was recognized not only by monoclonal antibodies that bind to the globular head domain but also by those that bind to the stalk domain. We also demonstrated that the data obtained by this assay correlate with the protection of an experimental vaccine in animal models.

## Abstract (precis)

Influenza virus HA-specific antibodies can be detected via the hemagglutination inhibition (HI) assay, the neutralization (NT) assay, and the enzyme-linked immunosorbent assay (ELISA). However, these assays have some drawbacks, including narrow dynamic range and the requirement for large amounts of sera.

## INTRODUCTION

Antibodies (Abs) are produced by plasma cells in response to non-self-antigens such as pathogen-associated molecules. Thus, the titer of specific Abs is a key parameter in the medical diagnosis of infectious disease.

Influenza virus infection causes acute respiratory disease and imposes a heavy burden on society. Effects are particularly severe in young children and the elderly (1). Annually, seasonal influenza epidemics cause 3 to 5 million severe cases in the world (2). Hemagglutinin (HA) and neuraminidase (NA) proteins are the major surface antigens (Ags) of the influenza virus and are targets for vaccine and antiviral drugs, respectively (3–5). HA-specific binding Abs and neutralizing Abs induced by various vaccines mediate protection against virus invasion.

The HA inhibition (HI) assay, virus neutralization (NT) assay, and enzyme-linked immunosorbent assay (ELISA), are widely used for epidemiologic and immunologic studies of influenza virus as well as in the evaluation of vaccine efficacy (6). However, these assays have drawbacks: the HI assay requires pretreatment of sera to remove endogenous nonspecific inhibitors, the NT assay requires a relatively large amount of serum, and the ELISA is not suitable for quantitative analysis of Ab levels because of the assay’s narrow dynamic range, given that the readout relies on absorbance. Moreover, preparation of Ags for ELISA is labor-intensive and expensive.

As an alternative to ELISA-based methods, the luciferase immunoprecipitation system (LIPS) was developed for the detection of Ag-specific Abs in sera from various animals (7, 8). LIPS requires the production of a protein comprising a reporter enzyme (luciferase) fused to an Ag of interest; this reagent is expressed from a plasmid vector in cultured cells. The luciferase-Ag fusion protein is recognized by Ag-specific Abs; the resulting Ag-Ab complexes are immobilized on protein A/G beads that recognize the fragment crystallizable (Fc) region of immunoglobulin G (IgG), and the Ag-specific Abs are quantified by measuring luminescence emitted by the luciferase-Ag fusion protein. This immunoassay represents a major improvement over ELISA technology in that LIPS is easily implemented to quantify the humoral response profile to various Ags in a universal format, providing a large amount of Ag while decreasing the time and effort needed to prepare highly purified Ags. Furthermore, LIPS has a low background with a broad dynamic range (spanning approximately 6 to 7 orders of magnitude). Luciferase-encoding genes derived from Gaussia princeps (copepod; GLuc), *Renilla* (jellyfish; RLuc), and Photinus pyralis (firefly; FLuc) have been employed for use in the LIPS assay (9–12). Among the luciferases, GLuc is in principle approximately 100-fold brighter than RLuc, although the former has a shorter half-life (about 2 min) (13). To overcome these issues, we adopted NanoLuciferase (NLuc) for use in the LIPS. Notably, NLuc is a small (19-kDa) enzyme derived from the deep-sea shrimp Oplophorus gracilirostris. The intensity of luminescence produced by NLuc is 150-fold higher than either FLuc or RLuc. Moreover, NLuc shows improved luminescence stability compared to the other luciferases, possessing a signal half-life of greater than 2 h (14).

HA proteins are known to assemble into a trimeric form via a process that depends on multiple posttranslational modifications, including disulfide bond formation and glycosylation (15). Several studies have shown that N-linked glycosylation of HA is important not only for the regulation of protein folding and stability but also for virus infectivity and antigenicity (16, 17). Immunogenicity of recombinant HA proteins is affected by both N-linked glycosylation and trimerization. Thus, the accurate measurement of HA-specific Abs requires the construction of recombinant HA proteins with native conformations.

In the present study, we developed recombinant influenza virus HA proteins fused to NLuc and used these fusion proteins for LIPS. We then employed the resulting assay, which we designated NanoLIPS, for monitoring the titer of Abs present in animal sera following either influenza virus infection or vaccination. Our data indicated that NLuc inserted at the C terminus of HA is modified by N-glycosylation and forms intact trimeric complexes that are recognized by monoclonal Abs (MAbs) that bind to the globular head or stalk domain of HA.

## RESULTS

### NLuc-based LIPS is more sensitive than GLuc-LIPS for detection of Abs in mouse sera.

**FIG 1:**
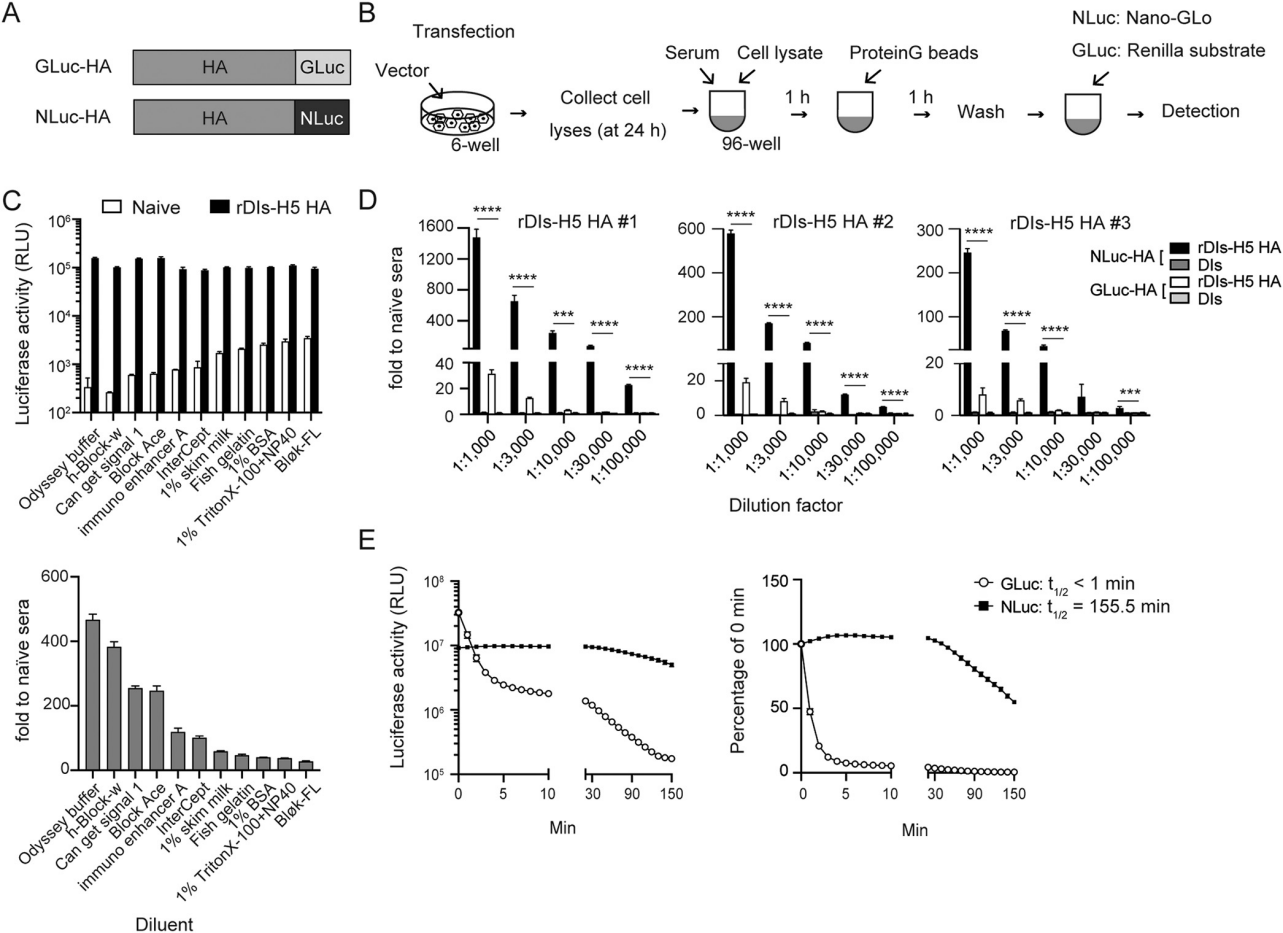
Comparison between NanoLuciferase (NLuc)- and *Gaussia* luciferase (GLuc)-based luciferase immunoprecipitation system (LIPS) assays. (A) Schematic representation of the recombinant GLuc-HA or NLuc-HA constructs used for LIPS assays. (B) Schematic of the procedure used for the NanoLIPS and GLIP assays. (C) Comparison of serum dilution buffers from several commercial sources. Raw values of relative luciferase activity (top) and respective signal-to-noise ratio (bottom) are shown (*n* = 3). (D) Detection of HA-binding antibodies by LIPS assay based on NLuc-HA versus GLuc-HA. Sera were collected from mice 3 weeks after inoculation with recombinant vaccinia virus expressing HA (rDIs H5 HA) derived from H5N1 virus (A/Bar-Headed Goose/Qinghai Lake/1A/05). Sera from three different vaccinated mice (high, middle, and low antibody titers) were diluted 1:1,000 to 1:100,000 (*n* = 3). The panel shows *P* values only for the comparison between NLuc-HA- and GLuc-HA-based assays performed using sera from mice vaccinated with rDIs-H5 HA. With the exception of rDIs-H5 HA #3 at the 1:30,000 dilution, assayed via NanoLIPS based on NLuc-HA, sera from mice vaccinated with rDIs-H5 HA showed significantly higher signal-to-noise ratios than did sera from control mice (vaccinated with DIs). Statistical analyses were performed by two-tailed one-way ANOVA with *post hoc* Tukey’s multiple-comparison tests (***, *P* < 0.001; ****, *P* < 0.0001). (E) Stability of NLuc versus GLuc signals. Raw luminescence activities (left), relative values (right), and the half-life (*t*_1/2_) of each signal are shown. Values are shown as means ± standard deviations (SD) (*n* = 3).

GLuc fusion proteins typically have been employed for the LIPS assay (GLIP). To compare the sensitivity of GLuc and NLuc reporters for the LIPS assay, we generated recombinant constructs encoding influenza virus H5N1 HA protein (A/Bar-Headed Goose/Qinghai Lake/1A/05, clade 2.2) fused with either GLuc (GLuc-H5 HA) or NLuc (NLuc-H5 HA) at the C terminus of the HA protein (Fig. 1A). Mouse sera were collected 3 weeks after vaccination with recombinant vaccinia virus expressing H5N1 HA protein (rDIs-H5 HA), and the titers of Ag-specific Abs were measured by either of the two different LIPS assays. NanoLIPS and GLIP were conducted as depicted schematically in Fig. 1B. To optimize conditions for diluting serum samples, we compared several blocking buffers from commercial sources. Unlike conventional blocking agents (e.g., bovine serum albumin [BSA] or skim milk) that yielded high background, Odyssey blocking buffer from LI-COR provided the highest signal-to-noise ratio (Fig. 1C). As shown in Fig. 1D, the ratio of values obtained for sera from immunized mice compared to that from naive mice (using 1,000-fold dilutions, i.e., 1:1,000) was at least 20-fold higher when assayed by NLuc-H5 HA-based LIPS than by GLIP. Furthermore, NLuc signal was readily detectable even at 1:100,000 dilutions of sera, in contrast to the signal from GLuc-H5 HA, which appeared to plateau at dilutions of 1:30,000 (Fig. 1D). No HA-specific Abs were detected from mice immunized with an empty vector control (DIs; Fig. 1D). We confirmed that the signal half-life of NLuc exceeded 150 min (Fig. 1E), whereas the GLuc signal decreased much more rapidly, exhibiting a half-life of less than 1 min (Fig. 1E). The greater stability of NLuc allowed us to establish an injector-free protocol for the measurement of luminescence. Together, these results demonstrate that this improved LIPS assay based on NLuc offers a highly sensitive and simple protocol for the detection of Ab titers.

For NanoLIPS, the NLuc fusion Ags were produced using HEK293FT cells. We expected that the quality of Ags might be affected by cell conditions. Therefore, to assess the batch-to-batch variability, we performed a NanoLIPS assay using three different batch lysates as Ags. Mouse sera were collected 3 weeks after inoculation with control DIs or rDIs-H5 HA and used to analyze the reproducibility of the NanoLIPS assay. No significant difference was observed in either the reactivity of H5 HA-specific Abs (see Fig. S1, left, in the supplemental material) or the background (Fig. S1, right) among the three different lysate batches.

10.1128/mSphere.01342-20.1 FIG S1 Effects of batch-to-batch variability in NanoLIPS antigen. NanoLIPS was performed using sera from mice 3 weeks after vaccination with rDIs-H5 HA (left) or control DIs (right). Statistical analysis was performed by two-tailed one-way ANOVA with *post hoc* Tukey’s multiple-comparison tests. Values are plotted as means ± SD (*n* = 3). ns, not significant (*P* ≥ 0.05). Download FIG S1, EPS file, 1.3 MB. Copyright © 2021 Honda et al. 2021 Honda et al. https://creativecommons.org/licenses/by/4.0/ This content is distributed under the terms of the Creative Commons Attribution 4.0 International license.

### C-NLuc HA is conformationally intact and undergoes glycosylation to form a trimer complex.

**FIG 2:**
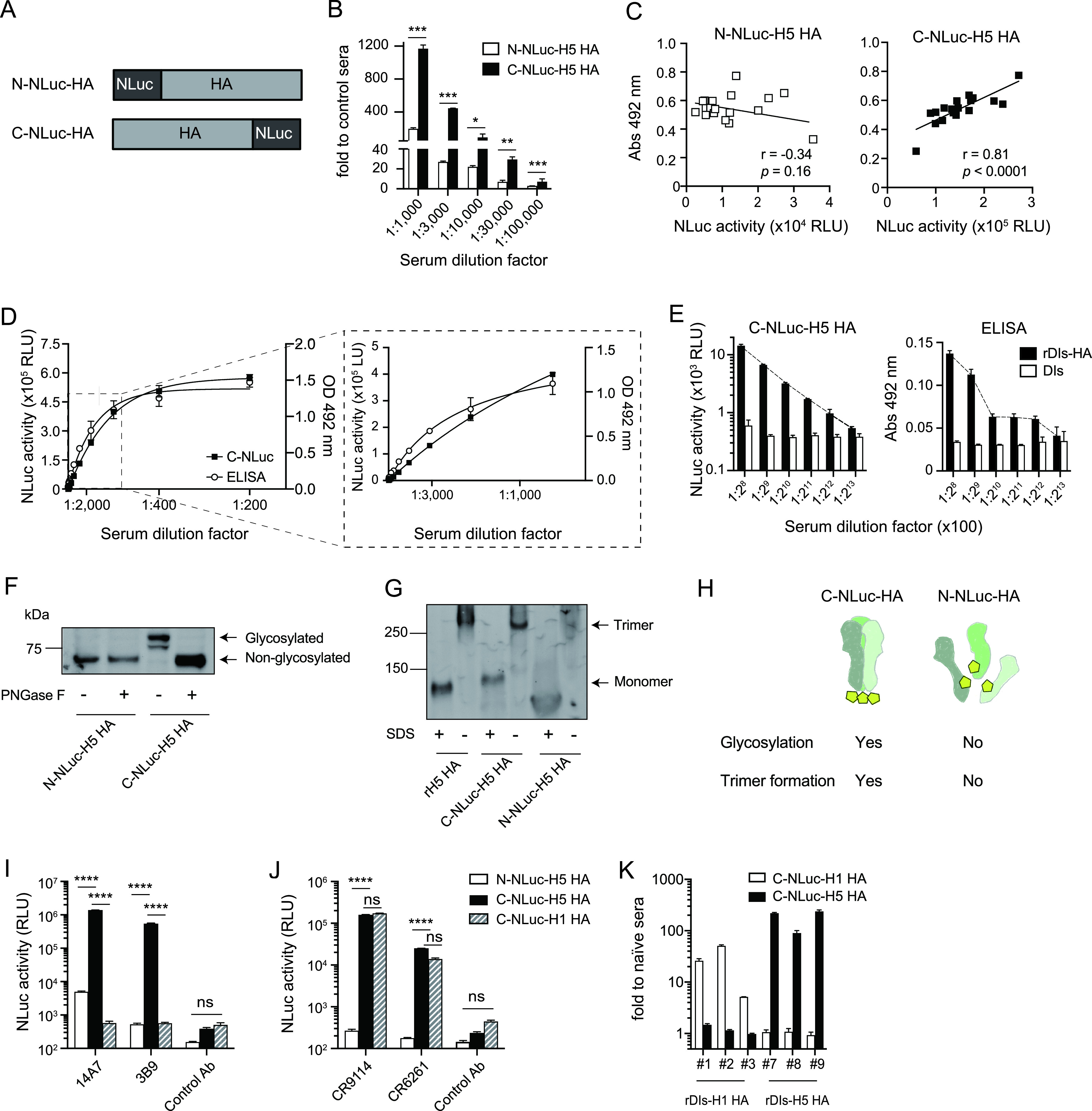
Comparison of effect of NLuc reporter inserted at the N terminus versus the C terminus of HA. (A) Schematic representation of N-terminal and C-terminal NLuc-HA constructs (N-NLuc-H5 HA and C-NLuc-H5 HA, respectively). (B) Detection of HA-binding antibodies by NanoLIPS based on N-NLuc- versus C-NLuc-H5 HA. Sera were collected from mice 3 weeks after inoculation with vaccinia virus expressing HA from H5N1 virus (A/Bar-Headed Goose/Qinghai Lake/1A/05) (*n* = 3). Comparisons were performed by two-tailed nonpaired Student’s *t* test (*, *P* < 0.05; **, *P* < 0.01; ***, *P* < 0.001 versus N-NLuc-H5 HA). (C) Correlation between ELISA and NanoLIPS assays. ELISA detection of HA-binding antibodies in mouse sera was conducted using C-terminally His-tagged recombinant H5 HA (A/Bar-Headed Goose/Qinghai Lake/1A/05). NanoLIPS assays based on N-NLuc-H5 HA or C-NLuc-H5 HA were performed using the same sera (*n* = 18). Abs, absorbance. (D and E) Detection limit of C-NLuc-H5 HA-based NanoLIPS versus ELISA. Sera from rDIs-H5 HA-vaccinated mice were subjected to serial dilution as indicated below the panels; assays were performed in triplicate (*n* = 3). OD, optical density. (F) Glycosylation of C-NLuc-H5 HA and N-NLuc-H5 HA was tested by digestion with peptide-N-glycosidase F (PNGase F) and electrophoresis by SDS-PAGE under reducing conditions. (G) Trimer complex formation by C-NLuc-H5 HA. Lysates of cells expressing C-NLuc-H5 HA, N-NLuc-H5 HA, or recombinant His-tagged H5N1 HA protein were electrophoresed by SDS-PAGE under reducing or nonreducing conditions and detected using anti-H5 HA 14A7 monoclonal antibody. (H) Schematic representation of N-glycosylation or trimerization conditions of each NLuc-tagged HA. (I and J) Monoclonal antibody against globular head domain of H5 HA (I) or stalk domain (J) of HA were detected by NanoLIPS based on N-NLuc-H5 HA versus C-NLuc-H5 HA, or C-NLuc-H1 HA (*n* = 3). ****, *P* < 0.0001; ns, not significant (*P* ≥ 0.05). (K) Verification of HA specificity of the NanoLIPS assay. Mice were inoculated with either rDIs-H5 HA or rDIs-H1-HA (derived from H1N1 virus). Four weeks after vaccination, mouse sera were collected and used for measuring HA-specific antibodies by NanoLIPS assay based on C-NLuc-HA derived from homologous or heterologous serotype HA (C-NLuc-H1HA or C-NLuc-H5 HA). Values are shown as means ± SD (*n* = 3).

We next constructed HA proteins fused with NLuc at the HA C-terminal (C-NLuc-H5 HA) or N-terminal (N-NLuc-H5 HA) end to determine whether the position of the NLuc insertion affected the assay (Fig. 2A). C-NLuc-H5 HA had a higher signal-to-noise ratio and better detection limit than N-NLuc-H5 HA when rDIs-H5 HA-vaccinated mouse sera were tested for HA-specific Abs (Fig. 2B). Given that ELISA is most typically used for detecting HA-specific Abs, we analyzed the correlation between the results of NanoLIPS and ELISA using a recombinant HA protein (rH5 HA) purified from recombinant vaccinia virus rVV-HA-infected RK13 cells as Ag. Data from NanoLIPS based on C-NLuc-H5 HA (*r* = 0.81, *P* < 0.0001), but not those from the assay based on N-NLuc-H5 HA (*r* = −0.34, *P* = 0.16), exhibited significant correlation with the results from ELISA (Fig. 2C). Furthermore, to determine the detection limit of each NanoLIPS method, serially diluted anti-rDIs-H5 HA serum samples were used for the assays. NanoLIPS demonstrated a broad linear range (from serum volume of 6.3 × 10^−2^ to 6.3 × 10^−5^ μl/reaction mixture, *r*^2^ = 0.99) (Fig. 2D), in contrast to the ELISA-based method (in which values plateaued at dilutions as low as 1:100,000 [1:100 × 2^10^]) (Fig. 2E). Thus, compared to conventional ELISA, NanoLIPS produced comparable results with a superior detection limit.

*N*-linked oligosaccharides on the HA protein play important roles in virus antigenicity, receptor binding, and infectivity (18, 19). Several reports have indicated that the sites of *N*-linked glycosylation within the HA stalk domain are well conserved among influenza virus strains and that glycosylation of HA contributes to the structural stability of the HA trimer (20). Therefore, we examined whether NLuc-HA proteins undergo the expected posttranslational modification and functional trimer complex formation under native conditions. Glycan attached to the globular head of HA is important for virus antigenicity (21); however, only C-NLuc-H5 HA (and not N-NLuc-H5 HA) exhibited glycosylation, as revealed by sensitivity to peptide-N-glycosidase F (PNGase F) treatment (Fig. 2F). Furthermore, native PAGE revealed that C-NLuc-H5 HA formed a trimer complex, as also seen with recombinant HA protein (rH5 HA) (Fig. 2G). Thus, these data indicated that the insertion of NLuc at the C terminus of HA does not impair the glycosylation profile or functional conformation of HA protein (Fig. 2H).

Next, we examined the binding of N- or C-NLuc-HA by MAbs that recognize the globular head or stalk domain of HA; this analysis used four different Abs known to target distinct HA epitopes or domains. Escape mutant analysis has indicated that MAbs 14A7 and 3B9 bind near the R156 and T183 residues, respectively, both of which are located in the globular head domain of H5 HA (22). CR9114 has been reported to bind an epitope near the hydrophobic pocket in HA2, thereby neutralizing both group 1 and group 2 influenza A viruses (23). CR6261 binds near the highly conserved stalk domain of group 1 influenza A virus (24). As shown in Fig. 2I and J, C-NLuc-H5 HA yielded significantly higher NLuc signal than N-NLuc-HA following immunoprecipitation with MAbs against either the globular head (14A7, 3B9) or the stalk domains (CR9114, CR6261) of HA. Similarly, C-NLuc-H1 HA showed comparable levels of NLuc signal following immunoprecipitation with MAbs against the stalk domain of HA but not with MAbs against the globular head domain of H5 HA. Furthermore, we compared the sensitivities of the C-NLuc-HA-based NanoLIPS assay and an ELISA that used the CR9114 MAb. NanoLIPS was able to detect 3 ng of Ab (Fig. S2A, left), a sensitivity that matched that of the ELISA (Fig. S2A, right). Both assays showed a significant correlation (Fig. S2B, *r* = 0.998, *P* < 0.0001). These data indicated that C-NLuc-HA has an intact conformation and can be used for detecting Abs that bind to the globular head domain of HA and also for detecting Abs that bind to the stalk domain of HA.

10.1128/mSphere.01342-20.2 FIG S2 Comparison of the sensitivities of the C-NLuc-HA-based NanoLIPS assay and an ELISA. (A) NanoLIPS (left) and ELISA (right) were performed using serial dilutions of the CR9114 monoclonal antibody (MAb). ELISA was conducted using C-terminally His-tagged recombinant H5 HA (A/Bar-Headed Goose/Qinghai Lake/1A/05) (*n* = 3). (B) ELISA and NanoLIPS assay were performed using serial dilutions of the CR9114 MAb. Linear regression curves were fitted to the data sets. Pearson’s correlation coefficient (r) and a two-tailed *P* value were calculated for the combination of ELISA and NanoLIPS data sets. Download FIG S2, EPS file, 1.2 MB. Copyright © 2021 Honda et al. 2021 Honda et al. https://creativecommons.org/licenses/by/4.0/ This content is distributed under the terms of the Creative Commons Attribution 4.0 International license.

However, influenza A viruses have at least 18 different subtypes of HA, meaning that a HA-specific Ab detection system would be required to distinguish among these HA serotypes. Therefore, we examined whether NanoLIPS could differentiate specific HAs from other influenza subtypes. NanoLIPS based on C-NLuc-H5 HA detected Abs from the sera of mice vaccinated with rDIs-H5 HA, whereas no HA-specific Abs were detected in the sera from rDIs-H1-vaccinated mice (Fig. 2K). Similar results were obtained using C-NLuc-H1 HA as an Ag (Fig. 2K). Thus, both NanoLIPS Ags could be immunoprecipitated only with the respective subtype-specific antisera. These results indicated that NanoLIPS can distinguish between H5 HA (avian HA) and H1 HA (seasonal HA).

### NanoLIPS assay can be used for detection of Abs from cynomolgus macaque and tree shrew.

**FIG 3:**
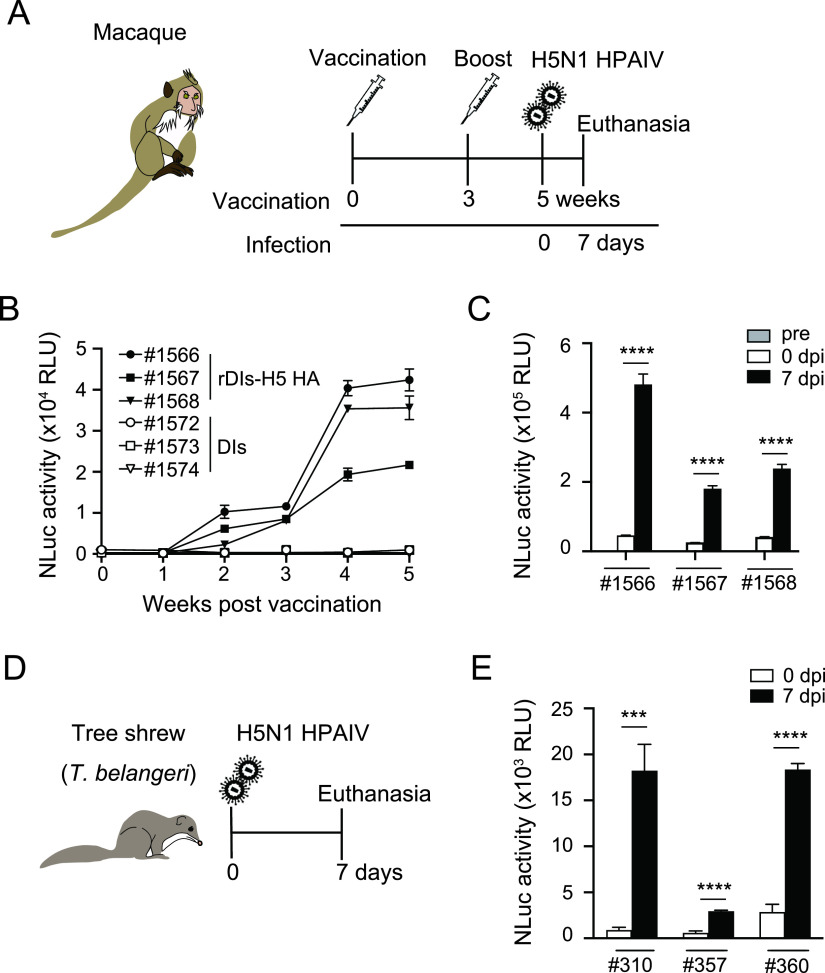
Application of NanoLIPS to detect HA antibodies in sera from macaques or tree shrews. (A) Schematic representation of the study design. Macaques were immunized intradermally (on the arm) with rDIs-H5 HA at 1.0 × 10^7^ PFU/animal at 0 weeks and boosted 3 weeks later at the same dose. Serum samples were collected every week after immunization. Five weeks after the initial vaccination, macaques were infected with 3.0 × 10^6^ PFU/animal of highly pathogenic avian influenza virus (HPAIV) A/whooper swan/Hokkaido/1/2008 H5N1. (B) Detection of HA antibodies by NanoLIPS based on C-NLuc-H5 HA at different time points postimmunization (*n* = 3). (C) NanoLIPS detection of HA antibodies in sera from macaques at 7 days after infection (dpi) with influenza virus. Statistical analysis was performed by two-tailed one-way ANOVA with *post hoc* Dunnett’s multiple-comparison tests (****, *P* < 0.0001 versus 0 dpi). (D) Schematic representation of the study design. Tree shrews were infected with 10^6^ PFU/animal of A/VietNam/UT3040/2004 H5N1 virus. Seven days after infection, tree shrews were euthanized, and serum samples were collected. (E) Specific antibodies measured by NanoLIPS assay using C-NLuc-H5 HA as antigen (*n* = 3). Statistical analysis was performed by two-tailed nonpaired Student’s *t* test (***, *P* < 0.001; ****, *P* < 0.0001). Values are shown as means ± SD.

Various experimental animal models have been developed for influenza challenge experiments (25–28). To evaluate whether NanoLIPS assay can be employed in these different models, we performed NanoLIPS assays using sera from two different species, cynomolgus macaque (Macaca fascicularis) and tree shrew (Tupaia belangeri). Macaque models have been used for investigating influenza pathogenesis and for evaluating the protective efficacy of influenza vaccines (29, 30). In the present study, macaques were immunized by two injections (administered 3 weeks apart) of 1 × 10^7^ PFU per injection of vaccinia virus (rDIs-H5 HA), followed by challenge with influenza virus 2 weeks after the booster (Fig. 3A). Ab responses against H5 subtype HA protein were measured using the NanoLIPS assay. After the first vaccination, the titer of HA-specific Abs increased gradually; the titer then increased approximately fourfold following the second vaccination (the boost, administered at 3 weeks) (Fig. 3B). Following the challenge with H5N1 highly pathogenic avian influenza virus (HPAIV), a large further increase (about 10-fold) in H5 HA-specific Ab titer was detected (Fig. 3C). To verify whether NanoLIPS is usable for detecting Ag-specific Ab responses in this animal model, we also investigated H5 HA-specific Ab responses by ELISA, using the same sera as those tested used in NanoLIPS; the ELISA employed recombinant H5 subtype HA protein as Ag. The data from NanoLIPS based on C-NLuc-H5 HA (Fig. 3B and C) exhibited a significant positive correlation with those from ELISA (Fig. S3A [*r* = 0.94, *P* < 0.0001] and S3B [*r* = 0.94, *P* < 0.0001]).

10.1128/mSphere.01342-20.3 FIG S3 Correlation analysis between ELISA and NanoLIPS. The data for NanoLIPS are from Fig. 3B (A) and Fig. 3C (B). Detection of H5 HA-specific antibodies by ELISA was performed using the same sera as those used for detection by NanoLIPS. ELISA was conducted using C-terminally His-tagged recombinant H5 HA (A/Bar-Headed Goose/Qinghai Lake/1A/05). Linear regression curves were fitted to the data sets. Pearson’s correlation coefficients (r) and two-tailed *P* values were calculated for the indicated combinations of data sets. Download FIG S3, EPS file, 1.2 MB. Copyright © 2021 Honda et al. 2021 Honda et al. https://creativecommons.org/licenses/by/4.0/ This content is distributed under the terms of the Creative Commons Attribution 4.0 International license.

The tree shrew is a small mammal that shares genetic similarities with primates (31, 32). We and other groups have demonstrated that tree shrews are susceptible to infection with both seasonal and avian influenza viruses (33–35). In the present study, serum samples from tree shrews infected with H5N1 influenza virus were collected 7 days after infection (Fig. 3D). Again, increases (fivefold or greater) in HA-specific Abs were detected in virus-infected tree shrew using a NanoLIPS assay based on C-NLuc-H5 HA (Fig. 3E). These results demonstrated that NanoLIPS can be used for detecting Ag-specific Abs in both macaque and tree shrew models.

### Evaluation of vaccine efficacies using NanoLIPS.

**FIG 4:**
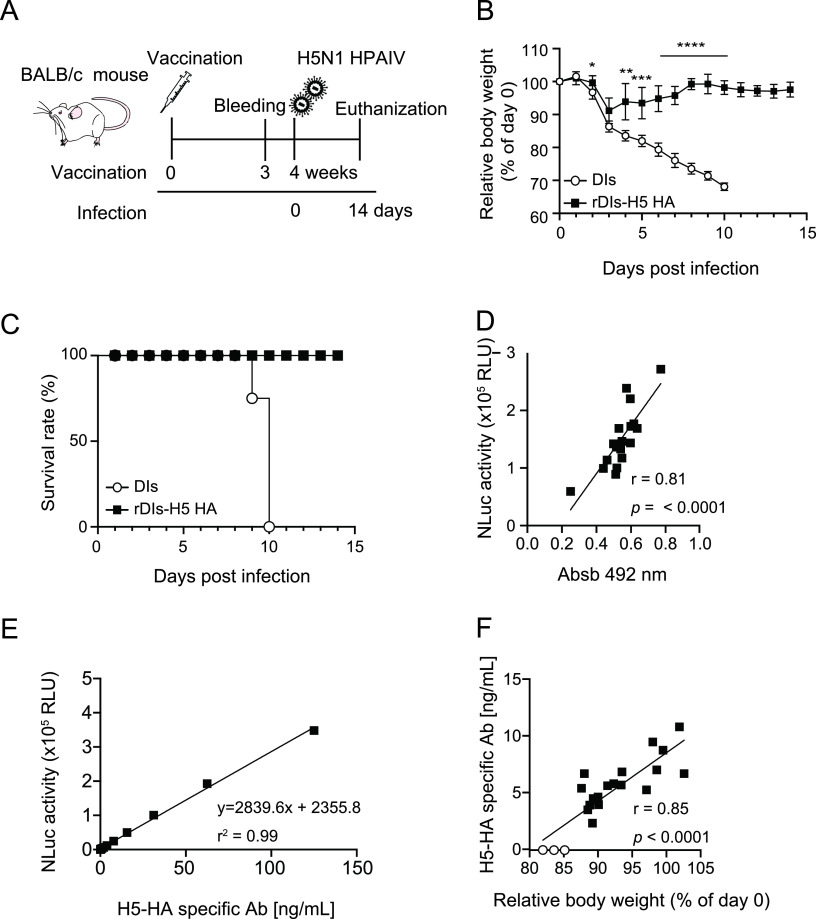
Correlation between NLuc activity and vaccine efficacy. (A) Schematic representation of the study design. Mice were immunized intradermally with rDIs-H5 HA vaccine at 10^7^ PFU/animal; serum samples were collected 3 weeks after vaccination. Four weeks after vaccination, mice were infected intranasally with 10^4^ PFU/animal of highly pathogenic avian influenza virus (HPAIV) A/whooper swan/Hokkaido/1/2008 H5N1. (B and C) Body weight change (B) and survival rate (C) were monitored following influenza virus challenge (for DIs, *n* = 3; for rDIs-H5 HA, *n* = 18). Statistical analysis was performed by two-tailed nonpaired Student's *t* test (*, *P* < 0.05; **, *P* < 0.01; ***, *P* < 0.001; ****, *P* < 0.0001 versus DIs control). Values are shown as means ± SD. (D) Correlation between ELISA and NanoLIPS. The data for NanoLIPS are from panel F. Detection of H5 HA-specific antibodies by ELISA was performed using the same sera as those assessed by NanoLIPS. ELISA was conducted using C-terminally His-tagged recombinant H5 HA (A/Bar-Headed Goose/Qinghai Lake/1A/05). Linear regression curves were fitted to the data sets, and Pearson’s correlation coefficients and two-tailed *P* values were calculated for the indicated combinations of data sets. Absb, absorbance. (E) The calibration curve of NanoLIPS was determined using serial dilutions of the CR9114 MAb. A linear regression curve was fitted to the data set. The coefficient of determination (*r*^2^) was calculated. (F) Correlation between relative body weight (3 days postinfection) and Ab concentration (3 weeks after vaccination). The NanoLuciferase activity has conversed to the Ab concentration based on the above linear regression curve (panel E). For DIs, *n* = 3 (open circles); for rDIs-H5 HA, *n* = 18 (closed squares). Linear regression curve was fitted to the data sets. Pearson’s correlation coefficient (*r*) and a two-tailed *P* value were calculated.

We next examined whether NanoLIPS can be used for testing vaccine efficacy. We evaluated the *in vivo* efficacy of rDIs-H5 HA against H5N1 HPAIV infection in a murine lethal infection model. Mice vaccinated with rDIs-H5 HA were infected with a lethal dose of H5N1 HPAIV and observed for body weight changes (Fig. 4A). All rDIs-H5 HA-vaccinated mice exhibited transient mild weight loss (a mean 9% [compared to baseline] at 3 days postinfection [dpi]), but these animals recovered rapidly, and all survived. In contrast, severe weight loss (>30% compared to baseline) and subsequent mortality (humane sacrifice) was seen in all control vaccinated mice by 10 dpi (Fig. 4B and C). We next evaluated correlations between the protective effect of vaccination and H5 HA-specific Ab titers (3 weeks after vaccination), as measured by NanoLIPS using C-NLuc-H5 HA; relative body weight (percentage of weight on day 0) was used as an indicator of vaccine efficacy. H5 HA-specific Ab responses were analyzed in antisera from the vaccinated mice by NanoLIPS assay and ELISA. As shown in Fig. 4D, the results obtained by NanoLIPS based on C-NLuc-H5 HA exhibited a significant positive correlation with the results obtained by ELISA (Fig. 4D, *r* = 0.81, *P* < 0.0001). We then quantified the H5 HA-specific Ab concentration based on the relative light unit (RLU; where one unit is defined as the number of luminometer units measured in 1 second) obtained from the NanoLIPS assay. First, the calibration curve was determined using CR9114, which is a commercially available and well-characterized H5 HA-specific MAb (*K*_*D*_ = 0.9 nM against H5 HA derived from A/Vietnam/1203/2004 H5) (23), which gave the linear regression (*r*^2^ = 0.99, *y* = 2839.6*x* + 2355.8) (Fig. 4E). The H5 HA-specific Ab concentration in antisera of vaccinated mice was then calculated by fitting the data to the linear equation. Notably, a correlation was detected between Ab concentration detected by C-NLuc-H5 HA-based NanoLIPS and relative body weight (*P* < 0.0001) (Fig. 4F). These results demonstrated that NanoLIPS using the C-NLuc-fused H5 HA protein is expected to be of use for evaluating vaccine efficacy.

## DISCUSSION

This study demonstrated that NanoLIPS offers a highly sensitive and simple technique for the detection of Ag-specific Abs present in sera from mouse, macaque, and tree shrew. In recent years, LIPS has been widely used for assessing immune responses in infectious diseases caused by HIV (36), Ebola virus (37), and influenza virus (38). In a previous study, a binding Ab titer against HA-1 of influenza virus, measured via *Renilla* luciferase-based LIPS, demonstrated a positive correlation with HI-determined titer. Moreover, LIPS could detect Ab titers that the HI assay could not detect (39). Thus, LIPS represents a powerful method for measuring Abs associated with various viral infections or vaccinations.

We further showed that NanoLIPS provides exceptional sensitivity compared to GLuc-based LIPS, allowing us to detect very low levels of HA-binding Abs in small aliquots of sera, under conditions in which the previous methods were unable to detect such Abs. Luminescence catalyzed by NLuc was at least 20-fold brighter than that from GLuc and exceptionally stable (half-life > 155 min), bypassing the need to use injector equipment for the Luc measurement. Moreover, NanoLIPS uses cell lysates expressing recombinant proteins as Ags. Because this technique bypasses the necessity of the purification step, NanoLIPS is a relatively simple method to generate Ags. The use of mammalian cells to express recombinant proteins allows for the generation of more suitable Ags that have undergone appropriate posttranslational modifications such as glycosylation.

We also showed that the NLuc fusion protein used for NanoLIPS had to be designed with caution, given that the antigenicity of the target protein was significantly affected by the position (N terminal versus C terminal) of the NLuc fusion. Careful assessment of NLuc positions at either the N or C terminus demonstrated that NanoLIPS based on C-NLuc, but not that based on N-NLuc, was capable of detecting MAbs that bind the globular head and stalk domains of influenza virus HA (Fig. 2I and J). We further found that C-terminal NLuc retains the intact conformation of HA, undergoing normal glycosylation and trimer formation, as shown by sodium dodecyl sulfate-polyacrylamide gel electrophoresis (SDS-PAGE) and native PAGE analysis (Fig. 2F and G). NanoLIPS results based on C-NLuc-H5 HA yielded data that exhibited a significant correlation with the protective effects of an rDIs-H5 HA vaccine (Fig. 4F). When vaccinated with rDIs-H5 HA, recipients produce a variety of Abs that bind to the globular head domain or stalk domain of HA. Among the strategies for influenza virus vaccine development, the stimulation of humoral responses against the globular head domain of HA is an important approach to preventing virus binding to the host cell or membrane fusion. On the other hand, Abs against the stalk domain of HA induce broad immunity and prevent infection with heterosubtypic influenza viruses (40). Thus, the induction of both types of Abs is crucial for preventing influenza virus infection. C-NLuc-H5 HA-based NanoLIPS was able to react with both Abs with specificity for the globular head domain of HA and those with specificity for the stalk domain. As shown in Fig. 2I and J, the binding sites and cross-reactivities of the detected Abs can be inferred using several serotypes of C-NLuc-HA as bait. Notably, the use of chimeric HAs (i.e., HAs comprising the head and stalk domains from distinct groups) (41) fused at their C termini with NLuc is expected to be very useful for binding site prediction of the detected Abs. Therefore, NanoLIPS, which facilitates Ag preparation, is expected to facilitate the evaluation of vaccine efficacy, supplementing or replacing the use of conventional methods such as ELISA and HI assay. It will be of great interest to test whether NanoLIPS can discriminate between Abs that recognize the globular head or the stalk domain of HA, potentially permitting more accurate analysis of the correlation of domain recognition and protective efficacy.

In the field of infectious disease, a variety of animal models are used. Because some viruses have a narrow host range, multiple animal models must be used in experiments. For instance, the hepatitis B virus exclusively infects humans, tree shrews, and chimpanzees (42, 43). Thus, an ideal immunological analysis should be applicable in a diverse range of animals. In the present study, we demonstrated that NanoLIPS can be used to detect Abs in serum samples derived from various experimental animal models, including the mouse, macaque, and tree shrew. Like other LIPS assays, NanoLIPS requires (in principle) only NLuc-tagged Ag and protein G beads for trapping the IgG present in small aliquots of sera. Thus, this assay system should be applicable for serum samples derived from various animal species, including wild animals, so long as the species’ immunoglobulins have affinity permitting binding to protein G. Further experiments will be necessary to evaluate the effectiveness of NanoLIPS in other experimental animals.

In this LIPS method, protein G magnetic beads do not permit distinction among specific classes of immunoglobulins. During actual immunization or viral infection, IgM-type Abs are produced in the primary Ab response and then undergo class switching to produce IgA or IgG Abs. Thus, the detection of IgM is often considered an indicator of recent infection, whereas the detection of IgG indicates previous exposure to an Ag or pathogen. Future work will be needed to establish immunoglobulin class-specific measurement methods.

Our data demonstrated that the NanoLIPS assay provides a simple technique for preparation of the assay Ag, yields data with a broad dynamic range, is highly sensitive, and is amenable to high-throughput analysis. This assay may be useful as a diagnostic tool for monitoring the history of infection and the effects of vaccination.

## MATERIALS AND METHODS

### Ethics statement.

This study was carried out in strict accordance with the *Guidelines for Animal Experimentation of the Japanese Association for Laboratory Animal Science* (44) and the recommendations in the *Guide for the Care and Use of Laboratory Animals* (45). All animal care and experimental procedures were performed according to the guidelines established by the Tokyo Metropolitan Institute of Medical Science Subcommittee on Laboratory Animal Care. All protocols were approved by the Committee on the Ethics of Animal Experiments of the Tokyo Metropolitan Institute of Medical Science.

### Generation of NLuc-antigen fusion-encoding constructs.

To construct NLuc-expressing vectors (pCAGGS-N-NLuc or pCAGGS-C-NLuc), the NLuc-encoding gene was amplified by PCR using Q5 polymerase (New England Biolabs, Ipswich, MA) with gene-specific primer sets; the pNL1.1 vector (Promega, Madison, WI) was used as the template for the NLuc-encoding sequences. The amplicons were digested with AsiSI and SbfI and were ligated into AsiSI-SbfI double-digested pCAGGS-Neo (Wako, Osaka, Japan) in which the simian virus 40 (SV40) poly(A) sequence had been replaced by the rabbit *β-globin* poly(A). Next, the gene for influenza virus HA (A/Bar-Headed Goose/Qinghai Lake/1A/2005 [H5N1-Clade2.2]) was amplified by PCR using gene-specific primers. The pCAGGS-N-NLuc and pCAGGS-C-NLuc vectors were linearized with restriction enzyme AsiSI or SbfI, respectively. The HA-encoding PCR fragments then were cloned into each vector using an In-Fusion HD Cloning kit (TaKaRa Bio, Shiga, Japan), yielding in-frame fusions of the NLuc- and HA-encoding open reading frames (ORFs).

### Production of GLuc or NLuc fusion proteins.

To express influenza virus HA fused to GLuc or NLuc, HEK293FT cells (ATCC, Manassas, VA) maintained in Dulbecco’s modified Eagle medium (Gibco) containing 10% fatal bovine serum (FBS) were transfected with purified plasmid DNA using Polyethylenimine MAX (Polysciences, Inc., Warrington, PA); the transfection was performed using 70 to 90% confluent cells grown in six-well plates. Twenty-four hours after transfection, cells were rinsed with phosphate-buffered saline without CaCl_2_ and MgCl_2_ [PBS(−)], and chilled *Renilla* lysis buffer (Promega) was distributed at 200 μl/well. Cells then were harvested with a cell scraper, and the lysate from each well was transferred to a 1.5-ml microfuge tube and centrifuged at 13,000 × *g* for 10 min at 4°C. The resulting supernatant was transferred to a fresh tube and stored at −80°C until use.

### Western blot analysis.

To analyze N-linked glycosylation of HA proteins, cell lysates containing NLuc-fused proteins were treated for 1 h at 37°C with peptide-N-glycosidase F (PNGase-F) (catalog no. P0704S; New England Biolabs, Inc.) and then separated by SDS-PAGE (10 μg total protein) using a SuperSep Ace 5 to 20% gel (Wako, Osaka, Japan) under reducing conditions, and the resulting gel was transferred to a polyvinylidene difluoride membrane. After blocking with Odyssey blocking buffer (LI-COR, Lincoln, NE), the membranes were incubated for 16 h at 4°C with rabbit anti-H5 HA polyclonal Ab (pep10; raised in our laboratory against a H5N1 HA peptide with the sequence DAAEQTRLYQNPTTYISVG) diluted 1:1,000 in the blocking buffer. After the membranes were washed three times with Tris-buffered saline containing 0.05% Tween 20 (TBS-T), they were incubated for 1 h at room temperature with anti-rabbit IgG IRDye 800CW (LI-COR) diluted 1:20,000 with the blocking buffer. After the membranes were washed three times with TBS-T, they were visualized using the Odyssey CLx infrared imaging system (LI-COR). Native PAGE was used to detect trimer complex formation by the HA proteins as described previously (46). Briefly, lysates suspended in native sample buffer (62.5 mM Tris-HCl [pH 6.8], 15% glycerol) were separated in running buffer (25 mM Tris and 192 mM glycine [pH 8.4] with or without 1% deoxycholate in the cathode and anode chambers, respectively). Mouse anti-H5 HA 14A7 MAb (developed in our laboratory) (22) and anti-mouse IgG IRDye 680RD (LI-COR) were used for detection of proteins separated by native PAGE.

### LIPS assay.

To optimize conditions for serum dilution, we compared several blocking buffers. The commercial blocking buffers tested included the following: Block Ace (catalog no. UKB80; Sumitomo Dainippon Pharma Co., Ltd. [formerly DS PHARMA Biomedical], Osaka, Japan), Blok FL (catalog no. WBAVDFL01; Millipore, USA, Burlington, MA), Can Get Signal-1 (catalog no. NKB-101S; Toyobo, Osaka, Japan), h-Block-w (catalog no. BCL-BKHW-01; Beacle, Kyoto, Japan), Immuno enhancer A (catalog no. 294-68601; FUJIFILM Wako Pure Chemical Corp., Osaka, Japan), Intercept (catalog no. 927-70001; LI-COR), and Odyssey blocking buffer (catalog no. 927-40000; LI-COR). NLuc or GLuc fusion proteins were diluted to 2 × 10^8^ RLU (where one unit is defined as the number of luminometer units measured in 1 second) per ml with Tris-based buffer (20 mM Tris-HCl [pH 7.5], 150 mM NaCl, 5 mM MgCl_2_, 1% Triton X-100, 25% glycerol) and transferred to a 96-well white plate (catalog no. 3789A; Corning, Corning, NY) at 50 μl (10^7^ RLU) per well. Mouse, tree shrew, or macaque sera diluted at 1:100 or 1:1,000 with Odyssey blocking buffer were added to the wells, and the plate was incubated for 1 h at room temperature with continuous rotation. Subsequently, 1 μl of SureBeads protein G magnetic beads (Bio-Rad, Hercules, CA) was added to each well, and the plate was further incubated for 1 h at room temperature with continuous rotation. The beads in each well were washed four times with PBS(+) [PBS(−) containing 0.9 mM CaCl_2_ and 0.5 mM MgCl_2_] -Tween 20 [PBS(+) containing 0.5% Tween 20] and once with PBS(+), and then suspended in 10 μl of PBS(+). An aliquot (10 μl) of Nano-Glo luciferase assay reagent (Promega) was added to each well, and the contents were mixed by pipetting. After 2 min of incubation at room temperature, the luciferase intensity was measured using an EnVision plate reader (PerkinElmer, Waltham, MA).

### Enzyme-linked immunosorbent assay (ELISA).

Ninety-six-well plates were precoated with 0.2 μg/ml recombinant C-terminal His-tagged H5 HA (derived from H5N1 A/Bar-Headed Goose/Qinghai Lake/1A/2005) protein in 50 mM carbonate buffer for 18 h at 4°C and then treated with blocking buffer (PBS containing 1% bovine serum albumin [BSA], 0.5% Tween 20, and 2.5 mM EDTA) for 2 h at room temperature. Diluted mouse sera were added to the wells, and the plates were incubated for 18 h at 4°C. After the wells were washed three times with PBS(−)-T [PBS(−) containing 0.05% Tween 20], horseradish peroxidase-conjugated anti-mouse IgG Ab (1:8,000) was distributed to the individual wells, and the plates were incubated for 1 h at room temperature. After the wells were washed five times, they were incubated with *o*-phenylenediamine for 30 min. The reactions were quenched by the addition of 2 M sulfuric acid, and the absorbance at 492 nm was measured using an EnVision plate reader.

### Generation of recombinant vaccinia virus.

Synthetic DNAs encoding the multibasic-site-deleted HA protein of H5N1 HPAIV (A/Bar-Headed Goose/Qinghai Lake/1A/05 [clade 2.2]) were purchased from Sloning BioTechnology (Puchheim, Germany). DNAs were amplified by PCR using specific primers with flanking restriction enzyme sites (HindIII). The resulting PCR fragments were digested with HindIII and ligated into a plasmid for recombination with the attenuated vaccine virus DIs strain (pUC/DIs-HA). pUC/DIs-HA was linearized with restriction enzymes and transfected into primary chicken embryo fibroblasts that had been infected with vaccinia virus strain DIs at a multiplicity of infection of 10 (47). After 24 h, the virus-cell mixtures were harvested and frozen at −80°C until required. Taking advantage of the fact that influenza virus HA protein agglutinates with guinea pig erythrocytes but vaccinia virus HA protein does not, HA-positive recombinant viruses that can recognize guinea pig erythrocytes were purified three times by plaque cloning, and the resulting virus stock was designated rDIs-H5 HA. Similarly, rDIs-H1 HA was generated by inserting HA gene derived from H1N1 pandemic influenza virus (A/California/07/2009) into the genome of strain DIs.

### Vaccination, infection, and serum sampling.

BALB/c mice (7-week-old females) were purchased from SLC (Hamamatsu, Japan). Mice were inoculated once with 10^7^ PFU per animal of rDIs-HA or DIs-empty (DIs); the latter served as controls. The inoculum was administered by intradermal (i.d.) injection at 10 sites of injection (5 μl/site) distributed across the lower back. Sera were collected from peripheral blood 3 weeks after vaccination. Four weeks after vaccination, mice were infected with 1.0 × 10^4^ PFU/animal of HPAIV (A/whooper swan/Hokkaido/1/08 [H5N1-Clade 2.3.2.1]). The body weights of mice were monitored, and the loss of 30% initial body weight was defined as the endpoint for euthanasia. Cynomolgus macaques (Macaca fascicularis) were obtained from Ina Research (Nagano, Japan). Macaques were inoculated i.d. by two separate injections with 1.0 × 10^7^ PFU/injection of rDIs-HA or DIs, administered 3 weeks apart. Sera were collected once weekly after vaccination. Five weeks after the initial inoculation (2 weeks after the second inoculation), macaques were infected with 3.0 × 10^6^ PFU/animal of HPAIV (A/whooper swan/Hokkaido/1/08 [H5N1-Clade2.3.2.1]); sera were collected at 7 dpi. Northern tree shrews (Tupaia belangeri) were purchased from the Kunming Institute of Zoology, Chinese Academy of Sciences (Kunming, China). The tree shrews were bred in the animal facilities of the Kagoshima University and Tsukuba Primate Research Center for experimental use. Tree shrews were infected with 1.0 × 10^6^ PFU/animal of influenza virus (A/VietNam/UT3040/2004 [H5N1-Clade1]); sera were collected at 7 dpi.

### Expression and purification of His-tagged recombinant HA.

The recombinant vaccinia virus expressed the multibasic-cyte-deleted HA protein derived from HPAIV A/Bar-Headed Goose/Qinghai Lake/1A/05 [H5N1-Clade2.2] (rVV-H5 HA). To express recombinant His-tagged influenza virus HA protein, RK13 cells (ATCC) maintained in minimum essential medium containing 5% FBS were infected with the rVV-H5 HA. Twenty-four hours after infection, cells were harvested with a cell scraper and stored at −80°C until use. Expressed His-tagged recombinant HA protein was purified using nickel-nitrilotriacetic acid (Ni-NTA) agarose (Qiagen, Venlo, Netherlands) according to the manufacturer’s protocol.

### Statistical analysis.

Statistical analyses were performed with Prism software (version 8.3.0; GraphPad, San Diego, CA). Results are reported as means ± standard deviations (SD). The *P* values were calculated using two-tailed nonpaired Student’s *t* tests for data from two groups or using one-way analysis of variance (ANOVA) or two-way ANOVA followed by *post hoc* Dunnett’s or Tukey’s multiple-comparison tests, as appropriate, for data from more than two groups/variables. *P* values of less than 0.05 were considered significant. The correlation coefficient (*r* value) was calculated using the corresponding function in Prism.
